# Clinical characteristics and prognostic significance of DNA methylation regulatory gene mutations in acute myeloid leukemia

**DOI:** 10.1186/s13148-023-01474-0

**Published:** 2023-03-29

**Authors:** Xiaoyan Xu, Hong Wang, Haohao Han, Yifang Yao, Xueqian Li, Jiaqian Qi, Chengsen Cai, Meng Zhou, Yaqiong Tang, Tingting Pan, Ziyan Zhang, Jingyi Yang, Depei Wu, Yue Han

**Affiliations:** 1 National Clinical Research Center for Hematologic Diseases, Jiangsu Institute of Hematology, The First Affiliated Hospital of Soochow University, No.188 Shizi Street, Suzhou, 215000 Jiangsu Province People’s Republic of China; 2 Institute of Blood and Marrow Transplantation, Collaborative Innovation Center of Hematology, Soochow University, Suzhou, People’s Republic of China; 3 Key Laboratory of Thrombosis and Hemostasis of Ministry of Health, Suzhou, People’s Republic of China; 4 State Key Laboratory of Radiation Medicine and Protection, Soochow University, Suzhou, People’s Republic of China; 5 Soochow Hopes Hematonosis Hospital, Suzhou, People’s Republic of China

**Keywords:** DNMT3A, IDH1, IDH2, TET2, Acute myeloid leukemia, Prognosis

## Abstract

**Background:**

DNA methylation is a form of epigenetic modification that regulates gene expression. However, there are limited data on the comprehensive analysis of DNA methylation regulated gene mutations (DMRGM) in acute myeloid leukemia (AML) mainly referring to DNA methyltransferase 3α (DNMT3A), isocitrate dehydrogenase 1 (IDH1), isocitrate dehydrogenase 2 (IDH2), and Tet methylcytidine dioxygenase 2 (TET2).

**Results:**

A retrospective study of the clinical characteristics and gene mutations in 843 newly diagnosed non-M3 AML patients was conducted between January 2016 and August 2019. 29.7% (250/843) of patients presented with DMRGM. It was characterized by older age, higher white blood cell count, and higher platelet count (P < 0.05). DMRGM frequently coexisted with FLT3-ITD, NPM1, FLT3-TKD, and RUNX1 mutations (P < 0.05). The CR/CRi rate was only 60.3% in DMRGM patients, significantly lower than in non-DMRGM patients (71.0%, P = 0.014). In addition to being associated with poor overall survival (OS), DMRGM was also an independent risk factor for relapse-free survival (RFS) (HR: 1.467, 95% CI: 1.030–2.090, P = 0.034). Furthermore, OS worsened with an increasing burden of DMRGM. Patients with DMRGM may be benefit from hypomethylating drugs, and the unfavorable prognosis of DMRGM can be overcome by hematopoietic stem cell transplantation (HSCT). For external validation, the BeatAML database was downloaded, and a significant association between DMRGM and OS was confirmed (P < 0.05).

**Conclusion:**

Our study provides an overview of DMRGM in AML patients, which was identified as a risk factor for poor prognosis.

**Supplementary Information:**

The online version contains supplementary material available at 10.1186/s13148-023-01474-0.

## Background

Acute myeloid leukemia (AML) is a malignancy characterized by molecular heterogeneity. The disease is marked by arrested cellular differentiation, over-proliferation of immature myeloid cells, and disruption of normal hematopoiesis, leading to severe bleeding, anemia, and infection [1]. In recent years, the advent of next-generation sequencing (NGS) technologies has facilitated the characterization and analysis of the genomic landscape in AML. This has led to the identification of several recurrent mutations, which have expanded our comprehension of the intricate molecular basis of AML [2–4].

A previous comprehensive series of leukemia genes revealed that approximately 96% of individuals with AML harbor at least one driver mutation [5]. Gene mutations can be classified into distinct subgroups based on their respective functions as follows: RNA splicing, DNA methylation, chromatin remodeling, transcription factors, activated signaling, cohesin complexes, tumor suppressors, and nuclear phospholipids [6–8]. In addition, DNA methylation has been found to occur early in the evolution of the disease [9, 10]. The extensive analysis of gene-wide DNA methylation patterns in hematopoietic lineage cells showed that DNA methylation is essential in hematopoiesis [11, 12]. Aberrant DNA methylation is frequently detected in hematologic malignancies, particularly in myelodysplastic syndromes (MDS) and AML [9].

Meanwhile, mutations in genes that regulate DNA methylation are primarily comprised of DNA methyltransferase 3α (DNMT3A), isocitrate dehydrogenase 1 (IDH1), isocitrate dehydrogenase 2 (IDH2), and Tet methylcytidine dioxygenase 2 (TET2). Over the past few decades, many studies have focused on these genes. DNMT3A mutations in AML have been shown to have an increased rate of disease recurrence and are strongly associated with poor prognosis, despite not added to the adverse risk classification in 2017 European Leukemia Network (ELN) risk stratification yet [13]. The recurrent mutations in IDH were IDH1^R132^, IDH2^R140^, and IDH2^R172^. IDH1/2 mutations have been proposed to be associated with pre-leukemic clones, serving as a valuable predictor for clinical relapse, and may be regarded as a reliable marker for minimal residual disease (MRD) monitoring [14–16]. Recently, novel inhibitors targeting the metabolic enzyme IDH1/2 have opened new avenues for treating these patients [17, 18]. Additionally, TET2 mutations are almost mutually exclusive with IDH1/2 mutations and the effect of TET2 mutations on prognosis is still debated [19, 20]. Nevertheless, current studies are limited by small sample sizes and a narrow focus on one or two mutations, thereby lacking an all-encompassing evaluation of these four DNA methylation regulatory genes [19, 21, 22].

In the present study, DNMT3A, IDH1, IDH2, and TET2 were referred to as DNA methylation regulatory gene mutations (DMRGM). Ryotokuji T et al. combined these DMRGM and conducted a DMRGM-based prognostic analysis study in 2016 covering 308 Japanese patients [23]. Therefore, we investigated the clinical features of Chinese patients (n = 843) and the relationship between such combined genotypes and clinical outcomes. We also discussed the efficacy of hypomethylating agents (HMA) in patients harboring DMRGM, which remained unaddressed in previous studies. Furthermore, external validation of the association between DMRGM and the prognosis was performed in the BeatAML database.

## Methods

### Patients

A total of 843 patients with newly diagnosed and receiving treatment for non-M3 AML at the First Affiliated Hospital of Soochow University between January 2016 and August 2019 were enrolled. Risk stratification of patients was classified according to the 2017 ELN risk criteria [24]. Informed consent was obtained from all patients before data collection. The study was approved by the Research Ethics Review Committee of the First Affiliated Hospital of Soochow University and was conducted following the Declaration of Helsinki.

### Data source

The Whole-Exome Sequencing (WES) data was downloaded from the BeatAML (http://www.vizome.org/aml), as well as the corresponding clinical and genetic information [4]. The propensity score matching was conducted in the cohorts from the BeatAML to confirm the consistency of enrolled patients according to the age, gender, and 2017 ELN risk stratification.

### Treatments

Induction chemotherapy primarily depended on the patients’ age and performance status, including standard and nonstandard first-line treatments. The standard first-line chemotherapy consisted of an IA/DA regimen (idarubicin 8 to 12 mg/m^2^ or daunorubicin 60 to 90 mg/m^2^, QD on days 1 to 3, and cytarabine 100 mg/m^2^, QD on days 1 to 7). For some patients with organ dysfunction or older age, chemotherapy regimens with low intensity were administered, including CAG (cytarabine 10 mg/m^2^, q12h on days 1 to 14; aclarubicin 7 mg/m^2^, QD on days 1 to 8; G-CSF 200 μg/m^2^, QD on days 1 to 14) or revised CAG (IAG: idarubicin 8 mg/m^2^, QD on days 1 to 3; cytarabine 10 mg/m^2^, q12h on days 1 to 14; G-CSF 200 μg/m^2^, QD on days 1 to 14; HAG: homoharringtonine 2 mg/m^2^, QD on days 1 to 7; cytarabine 10 mg/m^2^, q12h on days 1 to 14; G-CSF 200 μg/m^2^, QD on days 1 to 14; HCAG: homoharringtonine 2 mg/m^2^, QD on days 1 to 7 and CAG) with or without HMA. Consolidation chemotherapy consisted of intermediate or high-dose cytarabine-based regimens. More than half of the patients received hematopoietic stem cell transplantation (HSCT) after induction therapies.

Response to chemotherapy was evaluated after induction therapy for all but nine patients. Complete remission (CR) was defined as bone marrow blasts less than 5%, absence of circulating blasts, Auer rods or extramedullary disease, absolute neutrophil count ≥ 1.0*10^9^/L, and platelet count ≥ 100*10^9^/L. Incomplete hematologic recovery for all CR criteria was defined as CRi. The definition of partial remission (PR) was a reduction in myeloid cells from 5 to 25% and a reduction in myeloid cells of at least 50% before treatment. Overall response rate (ORR) included CR/CRi and PR. Relapse was defined as leukemic blasts ≥ 5% in bone marrow, reappearance of blasts in the blood, or the development of extramedullary disease.

### Conditioning regimens for HSCT

In accordance with 2017 ELN risk classification, most patients with intermediate or adverse risk underwent allogeneic HSCT, who received a myeloablative busulfan/cyclophosphamide-based conditioning regimens before transplantation. The specific schemes were as follows: busulfan 3.2 mg/kg/day, days − 7 to − 5; cyclophosphamide 1.8 g/m^2^/day, days − 4 to − 3. Patients receiving HLA-matched unrelated donor transplants and haploidentical donor transplants received rabbit anti-thymocyte globulin (ATG/thymoglobulin) (10 mg/kg total dose). Reduced-intensity conditioning was administered to older patients including fludarabine (30 mg/m^2^/day, days − 10 to − 6), cytarabine (1.5 g/m^2^/day, days − 10 to − 6), and busulfan (3.2 mg/kg/day, days − 5 to − 3).

### Mutation analyses

Comprehensive genetic mutational analyses of patients with ≥ 20% blasts in the bone marrow were performed. Genomic DNA was extracted using Purelink™ Genomic DNA Mini Kit (Invitrogen). The mutational status of forty-one genes was determined at an Ion S5 System (Thermo Fisher, Grand Island, NY, USA) and validated by Sanger sequencing.

### Statistical analysis

Overall survival (OS) was defined as the time from diagnosis to death (regardless of cause) or the last follow-up visit. Relapse-free survival (RFS) was calculated only for complete responders, from the date of achieving CR/CRi to the date of relapse or death, regardless of cause.

Categorical variables were described by frequency (percentage) and continuous variables by median (range). Rank sum and Fisher's exact test were carried out for categorical variables. Nonparametric analysis was conducted with the Mann–Whitney test. A pairwise comparison among multiple groups after Chi-square test was also conducted. Pearson correlation analysis was applied for correlation analysis. Kaplan–Meier curves were employed to assess survival. Univariate analyses were performed using log-rank tests while multivariate analyses with multivariate COX regression model tests. Variables with P values < 0.05 in univariate analyses were included in multivariate analysis. Differences with two-tailed P < 0.05 were considered statistically significant. Statistical analyses were performed using SPSS (version 26.0, IBM) and R version 4.2.0.

## Results

### Patient characteristics

This retrospective study enrolled eight hundred forty-three patients diagnosed with newly developed non-M3 AML. The cohort exhibited a median age of 43 years (range 9–78 years), with males comprising 52.7% of the study population. Most cases (73.2%) were classified in the intermediate risk group based on cytogenetic analysis. According to the 2017 ELN risk stratification, 42.3% of patients were classified within the favorable risk group, whereas 28.0% and 29.7% were assigned to the intermediate and adverse risk groups, correspondingly. Table 1 summarizes the baseline characteristics of all patients included in this study. A pairwise comparison among multiple groups was also conducted in Additional file 1: Table S1.

**Table 1:** Clinical characteristics between patients with and without DMRGM

Variables	Total n = 843 (%)	DMRGM (+) n = 250 (%)	DMRGM (-) n = 593 (%)	P value
Gender				0.686
Male	444 (52.7)	129 (51.6)	315 (53.1)	
Female	399 (47.3)	121 (48.4)	278 (46.9)	
Age, years				** < *0.001***
Median (Range)	43 (9–78)	49 (10–78)	39 (9–74)	
WBC count, *10^9^/L				***0.044***
Median (Range)	14.2 (0.1–406.9)	18.1 (0.5–383.8)	13.8 (0.1–406.9)	
Hemoglobin, g/L				0.979
Median (Range)	85 (33–171)	85 (34–140)	85 (33–171)	
Platelet count, *10^9^/L				** < *0.001***
Median (Range)	42 (2–713)	55 (4–713)	37 (3–376)	
Karyotype				** < *0.001***
Favorable	102 (12.1)	8 (3.2)	94 (15.9)	
Intermediate	617 (73.2)	208 (83.2)	409 (69.0)	
Adverse	91 (10.8)	19 (7.6)	72 (12.1)	
Unknown	33 (3.9)	15 (6.0)	18 (3.0)	
2017 ELN				***0.014***
Favorable	357 (42.3)	99 (39.6)	258 (43.5)	
Intermediate	236 (28.0)	87 (34.8)	149 (25.1)	
Adverse	250 (29.7)	64 (25.6)	186 (31.4)	
Induction therapy				***0.001***
Standard therapy	477 (56.6)	117 (46.8)	360 (60.7)	
Low intensity	308 (36.5)	114 (45.6)	194 (32.7)	
Others	58 (6.9)	19 (7.6)	39 (6.6)	
Response to first induction therapy^a^				***0.014***
CR/CRi	566 (67.9)	149 (60.3)	417 (71.0)	
PR	102 (12.2)	33 (13.4)	69 (11.8)	
NR	147 (17.6)	58 (23.5)	89 (15.2)	
Early death	19 (2.3)	7 (2.8)	12 (2.0)	
Transplant				0.079
Yes	474 (56.2)	129 (51.6)	345 (58.2)	
No	369 (43.8)	121 (48.4)	248 (41.8)	

DMRGM: DNA methylation regulatory gene mutations; WBC: white blood cell; 2017 ELN: 2017 European Leukemia Network; CR: complete remission; CRi: complete remission with incomplete hematological recovery; PR: partial remission; NR: no response

^a^Response to first induction therapy was unknown in 9 patients. A P value of less than 0.05 is indicated in italics and bold

**Fig. 1:**
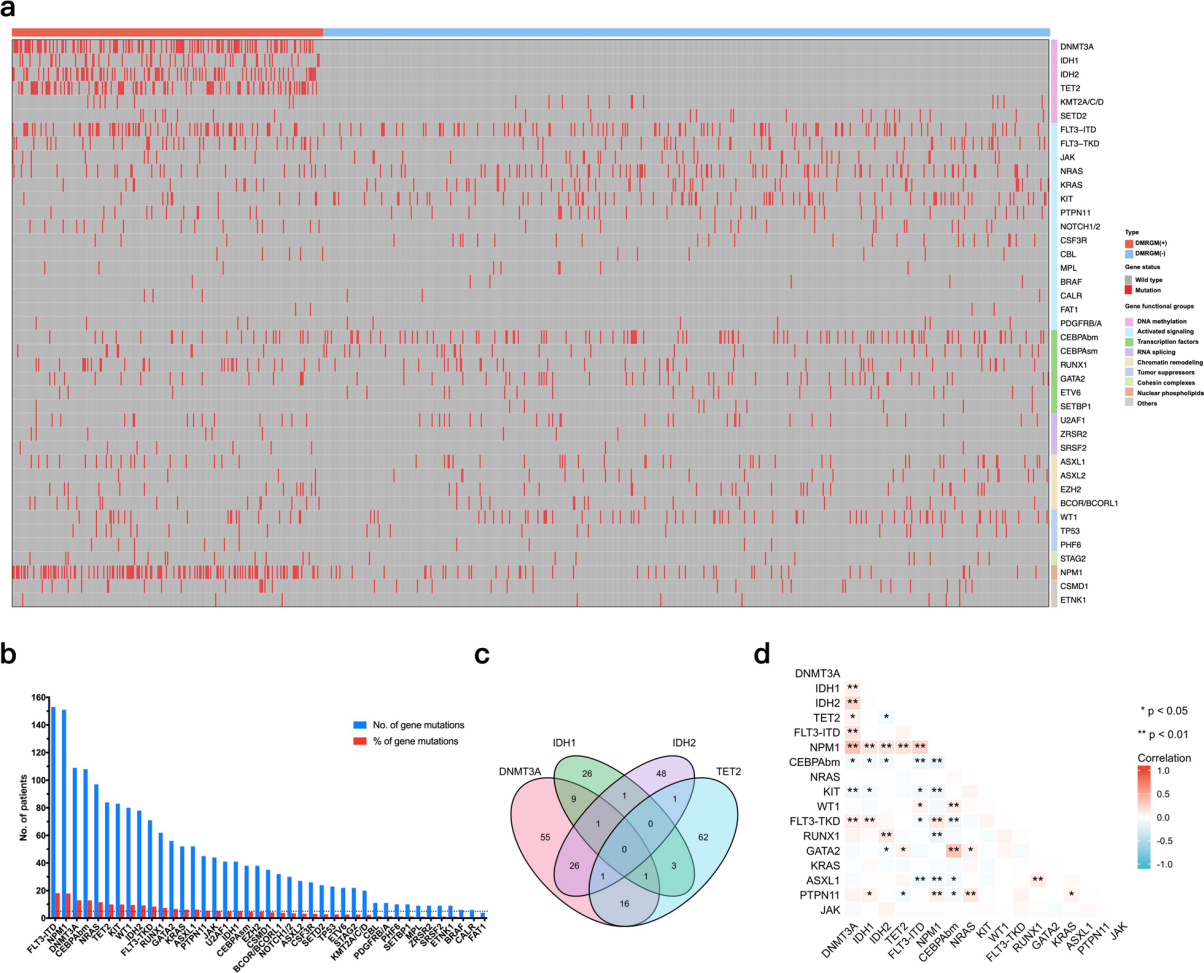
Genomic landscape of acute myeloid leukemia (AML). **a** Gene mutations assigned to functional groups with AML. **b** Frequency of analyzed genetic mutations in AML. **c** Venn diagram of frequency and overlap of DNA methylation regulatory gene mutations (DMRGM). **d** Genome distribution and collinearity of DMRGM

The mutations assigned to the functional group of new-onset AML in this study are shown in Fig. 1a. In addition, 731 of 843 patients (86.7%) experienced ≥ 1 mutation, the most frequent of which were FLT3-ITD mutation (153, 18.2%), NPM1 mutation (151, 17.9%) and DNMT3A mutation (109, 12.9%) (Fig. 1b). The following statistical analysis excluded the frequency of mutations detected in ≤ 5% of cases.

### Characteristics and overlapping gene mutations of patients with DMRGM

Of the 843 patients, 250 (29.7%) had DMRGM, including 191 (22.7%) with a single mutation (DMRGM-1), 56 (6.6%) with a double mutation (DMRGM-2), and 3 (0.4%) with a triple mutation (DMRGM-3). No case had a common mutation in all four genes (Fig. 1c). Patients with DMRGM were more frequently found to be older, along with higher white blood cell counts and platelet counts (P < 0.05). According to the 2017 ELN risk stratification, most DMRGM patients were classified in the favorable or intermediate risk group (39.6% favorable risk and 34.8% intermediate risk, P = 0.014) (Table 1). The clinical characteristics of each DMRGM (DNMT3A^mut^ vs. DNMT3A^wt^, IDH1^mut^ vs. IDH1^wt^, IDH2 ^mut^ vs. IDH2 ^wt^, and TET2 ^mut^ vs. TET2 ^wt^) patient are shown in Additional file 1: Table S2.

Moreover, we explored the overlapping gene mutations in DMRGM patients (Additional file 1: Table S3). DMRGM was preferentially associated with FLT3-ITD, NPM1, FLT3-TKD, and RUNX1 (P < 0.05) mutations but not with KIT or biallelic CEBPA mutations (P < 0.05). The covariation of intra-gene hotspots is shown in Fig. 1d.

### Risk factors affecting OS and RFS

The results of univariate and multivariate analyses of risk factors for OS are shown in Table 2. DMRGM were associated with poor OS in univariate analysis, but not an independent prognostic factor in multivariate analysis. Older age (HR: 1.322, 95% CI: 1.002–1.745, P = 0.048), higher white blood cell count (HR: 1.390, 95% CI: 1.018–1.898, P = 0.038), intermediate/adverse karyotype (int vs fav: HR: 1.778, 95% CI: 1.185–2.666, adv vs fav: HR: 3.600, 95% CI: 2.285–5.672, P < 0.001), and FLT3-ITD mutations (HR: 1.433, 95% CI: 1.095–1.876, P = 0.009) were confirmed to be independent risk factors for OS in multivariate analysis. In addition, biallelic CEBPA mutations (HR: 0.463, 95% CI: 0.303–0.709, P < 0.001) and receipt of hematopoietic stem cell transplantation (HR: 0.265, 95% CI: 0.208–0.338, P < 0.001) were identified as variables that predict a longer OS.

**Table 2:** Univariate and multivariate analyses of risk factors for OS

Variables	Univariate analysis			Multivariate analysis	
	No. of patients	3-year OS (%)	P value	HR (95%CI)	P value
Gender			0.543		
Male	444	61.2			
Female	399	59.9			
Age, years			** < *0.001***	1.322 (1.002–1.745)	***0.048***
< 60	739	64.6			
≥ 60	104	32.7			
WBC count, *10^9^/L			***0.002***	1.390 (1.018–1.898)	***0.038***
< 100	742	62.6			
≥ 100	101	45.8			
Hemoglobin, g/L			0.618		
< 100	581	59.9			
≥ 100	262	62.1			
Platelet count, *10^9^/L			0.848		
< 100	686	61.0			
≥ 100	157	58.7			
Karyotype^a^			** < *0.001***		** < *0.001***
Favorable	102	73.1		1	
Intermediate	617	62.4		1.778 (1.185–2.666)	
Adverse	91	38.7		3.600 (2.285–5.672)	
2017 ELN			** < *0.001***		
Favorable	357	74.9			
Intermediate	236	53.0			
Adverse	250	47.3			
HSCT			** < *0.001***	0.265 (0.208–0.338)	** < *0.001***
Yes	474	77.2			
No	369	39.1			
DMRGM	250	52.3	***0.001***	1.143 (0.904–1.444)	0.263
FLT3-ITD^Mut^	153	47.5	***0.001***	1.433 (1.095–1.876)	***0.009***
NPM1^Mut^	151	59.7	0.807		
DNMT3A^Mut^	109	40.0	** < *0.001***		
CEBPA^bm^	108	79.3	** < *0.001***	0.463 (0.303–0.709)	** < *0.001***
NRAS^Mut^	97	63.0	0.482		
TET2^Mut^	84	53.7	0.295		
KIT^Mut^	83	60.8	0.719		
WT1^Mut^	80	55.3	0.524		
IDH2^Mut^	78	54.8	0.375		
FLT3-TKD^Mut^	71	61.5	0.788		
RUNX1^Mut^	62	57.5	0.317		
GATA2^Mut^	56	59.9	0.827		
KRAS^Mut^	52	50.1	0.062		
ASXL1^Mut^	52	56.8	0.497		
PTPN11^Mut^	45	50.5	0.084		
JAK^Mut^	44	63.4	0.726		
IDH1^Mut^	41	60.9	0.505		

OS: overall survival; WBC: white blood cell; 2017 ELN: 2017 European Leukemia Network; HSCT: hematopoietic stem cell transplantation; DMRGM: DNA methylation regulatory gene mutations. A P value of less than 0.05 is indicated in italics and bold

^a^Karyotype data is unknown form 33 patients

Regarding RFS, 780 patients who achieved CR/CRi were included in univariate and multivariate Cox proportional regression analyses (Table 3). According to univariate analysis, older age (≥ 60 years), intermediate/adverse karyotype, intermediate/adverse risk of 2017 ELN, not having received HSCT, gene mutation status (NPM1^wt^, CEBPA^wt^, and PTPN11^mut^), and DMRGM were significantly associated with shorter RFS (P < 0.05). Multivariate analysis showed that older age (HR: 1.715, 95% CI: 1.109–2.652, P = 0.015), intermediate/adverse karyotype (int vs fav: HR: 2.396, 95% CI: 1.202–4.775, adv vs fav: HR: 2.953, 95% CI: 1.323–6.591, P = 0.024), biallelic CEBPA mutation (HR: 0.530, 95% CI: 0.295–0.952, P = 0.034), and DMRGM (HR: 1.467, 95% CI: 1.030–2.090, P = 0.034) were independent prognostic factors for RFS.

**Table 3:** Univariate and multivariate analyses of risk factors for RFS

Variables	Univariate analysis			Multivariate analysis	
	No. of patients	3-year RFS (%)	P value	HR (95%CI)	P value
Gender			0.072		
Male	412	76.5			
Female	368	81.1			
Age, years			** < *0.001***	1.715(1.109–2.652)	***0.015***
< 60	692	80.3			
≥ 60	88	63.0			
WBC count, *10^9^/L			0.130		
< 100	690	79.4			
≥ 100	90	68.3			
Hemoglobin, g/L			0.617		
< 100	534	78.7			
≥ 100	246	78.7			
Platelet count, *10^9^/L			0.520		
< 100	632	79.3			
≥ 100	148	76.0			
Karyotype^a^			***0.012***		***0.024***
Favorable	96	90.0		1	
Intermediate	575	77.8		2.396(1.202–4.775)	
Adverse	80	67.8		2.953(1.323–6.591)	
2017 ELN			***0.005***		
Favorable	344	84.4			
Intermediate	216	73.5			
Adverse	220	72.4			
HSCT			***0.014***	0.769(0.533–1.108)	0.158
Yes	468	81.4			
No	312	74.2			
DMRGM	227	72.0	***0.002***	1.467(1.030–2.090)	***0.034***
FLT3-ITD^Mut^	131	78.2	0.545		
NPM1^Mut^	140	72.7	***0.027***	0.966(0.622–1.498)	0.876
DNMT3A^Mut^	96	63.4	** < *0.001***		
CEBPA^bm^	105	88.2	***0.016***	0.530(0.295–0.952)	***0.034***
NRAS^Mut^	92	79.0	0.915		
TET2^Mut^	75	68.0	***0.023***		
KIT^Mut^	81	76.1	0.373		
WT1^Mut^	71	80.4	0.995		
IDH2^Mut^	71	73.5	0.451		
FLT3-TKD^Mut^	65	76.4	0.813		
RUNX1^Mut^	58	73.0	0.355		
GATA2^Mut^	53	85.3	0.255		
KRAS^Mut^	44	72.5	0.701		
ASXL1^Mut^	47	84.4	0.148		
PTPN11^Mut^	42	70.6	***0.048***	1.610(0.878–2.950)	0.124
JAK^Mut^	42	73.1	0.526		
IDH1^Mut^	39	80.0	0.979		

RFS: relapse-free survival; WBC: white blood cell; 2017 ELN: 2017 European Leukemia Network; HSCT: hematopoietic stem cell transplantation; DMRGM: DNA methylation regulatory gene mutations. A P value of less than 0.05 is indicated in italics and bold

^a^Karyotype data is unknown form 29 patients

### Prognostic significance of DMRGM on response and survival

**Fig. 2:**
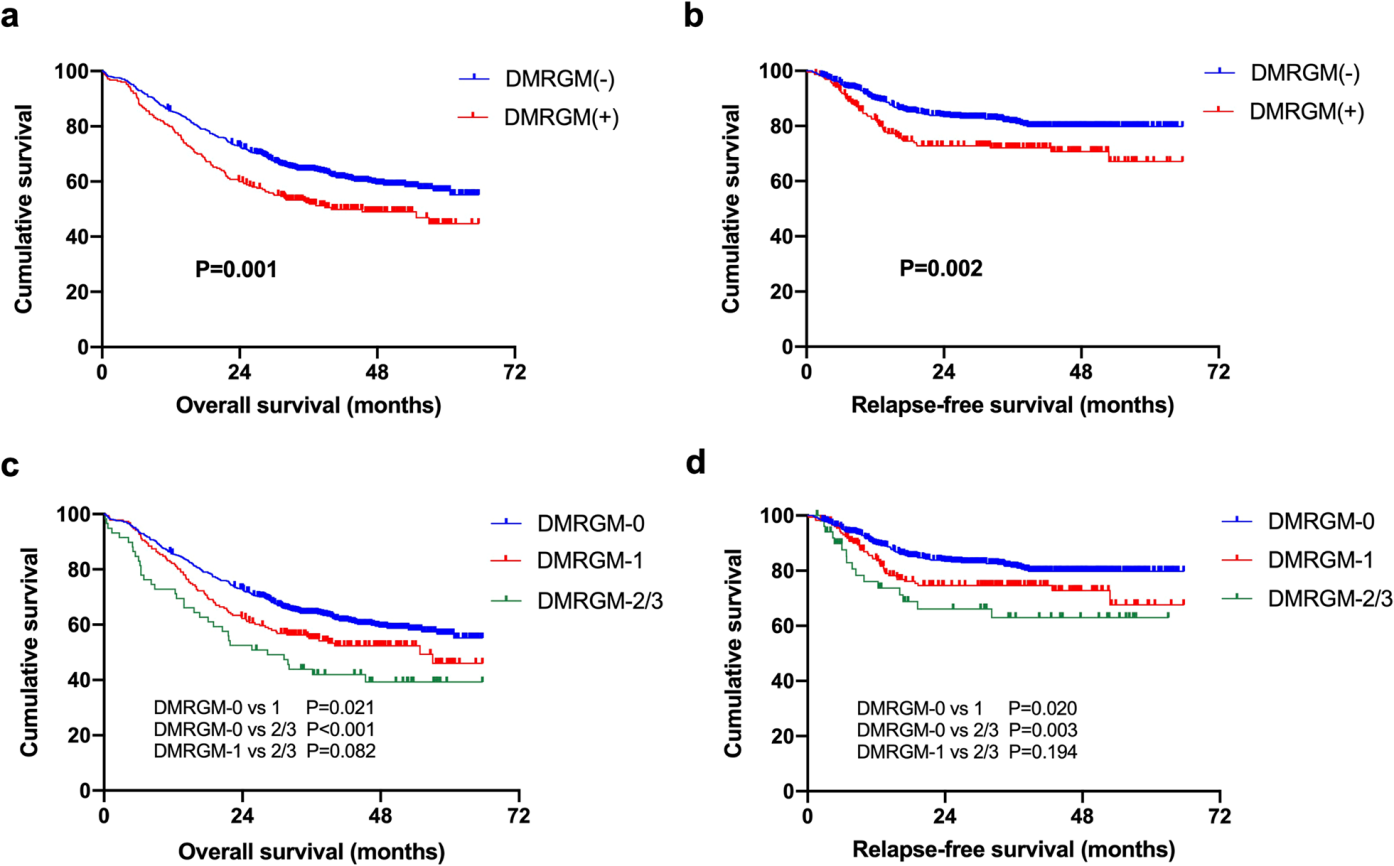
Overall survival (OS) and relapse-free survival (RFS) in AML patients with or without DNA methylation regulatory gene mutations (DMRGM). **a** OS in all patients based on DMRGM. **b** RFS in all patients based on DMRGM. **c** OS in all patients with different number of DMRGM. **d** RFS in all patients with different number of DMRGM

As shown in Table 1, DMRGM had a significant effect on the response to induction chemotherapy. The CR/CRi rate of patients with DMRGM was only 60.3%, which was substantially lower than that of patients without DMRGM (71.0%, P = 0.014). To further limit the confusion of age or induction chemotherapy regimens on the assessment of efficacy, subgroup analyses were performed, and similar results were observed that patients with DMRGM had a lower CR/CRi rate in subgroups of patients younger than 60 years (61.6% vs. 71.7%, P = 0.013) or with low-intensity induction chemotherapy (59.6% vs. 74.7%, P = 0.019). DMRGM was also confirmed as a prognostic factor by our results. 3-year OS rate was significantly lower in patients with DMRGM than those without DMRGM (52.3% vs. 64.1%, P = 0.001) (Fig. 2a). It was also an independent risk factor for RFS (P = 0.034) (Fig. 2b).

In addition, we investigated the effect of the number of DMRGM on prognosis. Notably, the findings of the survival analysis pertaining to the number of DMRGMs revealed a significant reduction in the 2-year OS rate and 2-year RFS rate among patients with DMRGM-2/3 compared to those with DMRGM-1 (52.5% vs. 62.3%, 66.1% vs. 74.6%) (Fig. 2c and Fig. 2d). While the observed difference did not attain statistical significance, a notable trend was discernible, indicating that future studies may potentially establish significant findings. For the current analysis, fifty-six patients had mutations in two genes (DMRGM-2), and only three had DMRGM-3. Therefore, we explored the relationship between the different combinations of DMRGM and the prognosis of DMRGM-2 patients. As a result, no significant difference in prognosis was established between the various combinations of DMRGM-2 (Additional file 1: Figure S1). To conclude, DMRGM has a discernible impact on both the response to chemotherapy and OS. Notably, individuals harboring two or more mutated genes associated with DMRGM tend to have poorer OS outcomes compared to those with a single DMRGM mutation.

### Prognostic impact of DMRGM on different 2017 ELN risk groups

**Fig. 3:**
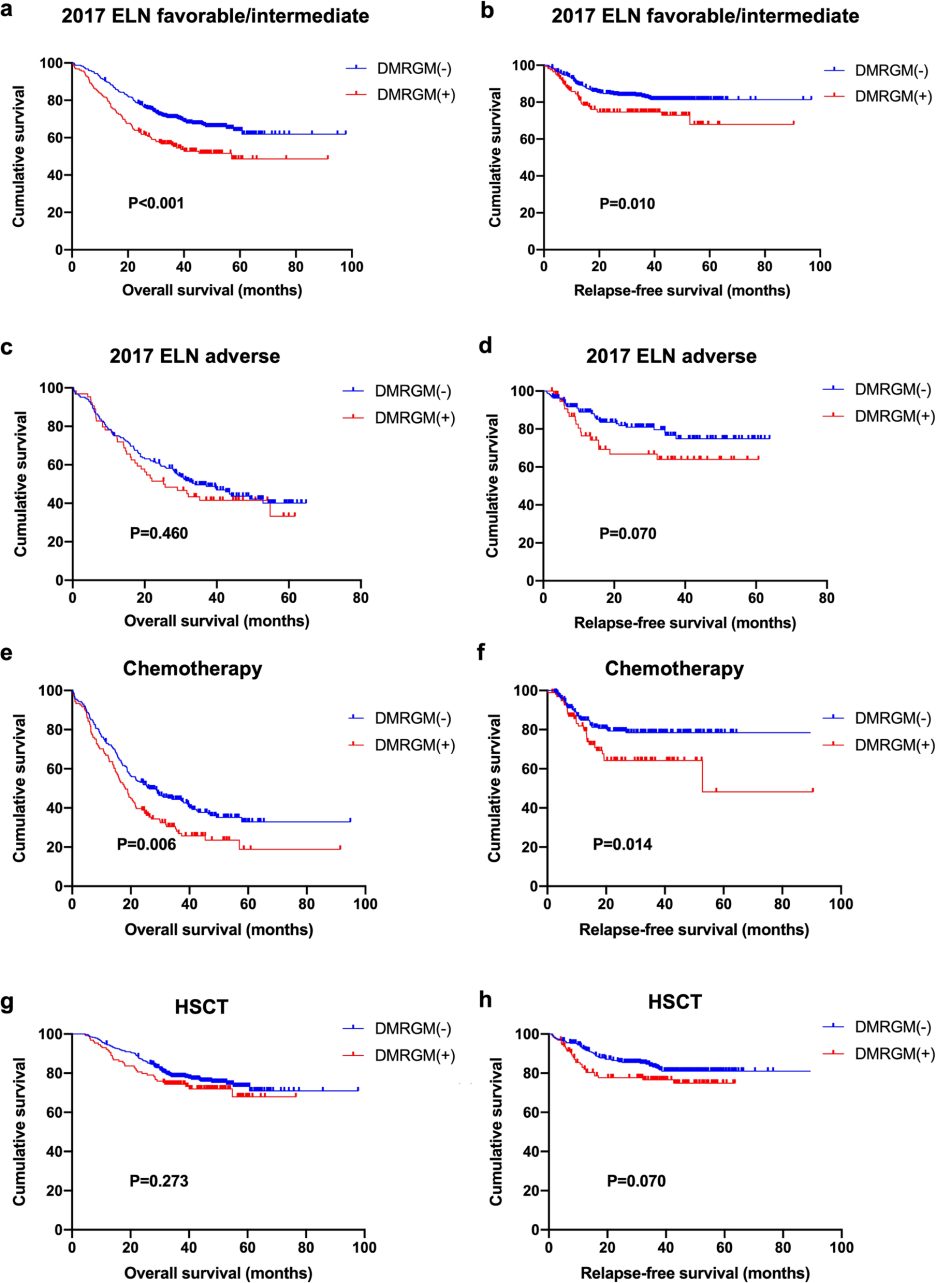
Subgroup analysis of overall survival (OS) and relapse-free survival (RFS) in AML patients according to 2017 ELN risk stratification and different consolidation therapy. **a** OS for patients in favorable/intermediate risk group based on DNA methylation regulatory gene mutations (DMRGM). **b** RFS for patients in favorable/intermediate risk group based on DMRGM. **c** OS for patients in adverse risk group based on DMRGM. **d** RFS for patients in adverse risk group based on DMRGM. **e** OS for patients receiving chemotherapy based on DMRGM. **f** RFS for patients receiving chemotherapy based on DMRGM. **g** OS for patients receiving HSCT based on DMRGM. **h** RFS for patients receiving HSCT based on DMRGM

According to our results, patients with DMRGM were more frequently categorized as favorable or intermediate risk group (70.3%) according to the 2017 ELN risk stratification. Therefore, we performed further analysis to assess the prognostic impact of DMRGM on patients according to the 2017 ELN risk stratification. As shown in Fig. 3a, in the favorable/intermediate risk group, patients with DMRGM had poorer OS (P < 0.001). Similar results were observed in RFS, where DMRGM was associated with poor prognosis (P = 0.010) (Fig. 3b). However, there was no statistically significant difference in OS and RFS between the adverse risk groups in patients with and without DMRGM (Fig. 3c and Fig. 3d). In conclusion, DMRGM indicates a poor prognosis for patients in the favorable/intermediate risk group.

### Efficacy of HSCT as consolidation for patients with DMRGM

Consistent with our results, hematopoietic stem cell transplantation significantly improved the prognosis of AML. OS and RFS were prolonged in patients who received HSCT after remission compared with those who received chemotherapy consolidation (Additional file 1: Figure S2). Stratified analysis showed that DMRGM was a valid predictor of poor OS and RFS in the chemotherapy subgroup (Fig. 3e, f). However, for patients receiving HSCT, DMRGM was not identified as a risk factor for OS and RFS (Fig. 3g, h). These results suggest that hematopoietic stem cell transplantation can significantly improve the prognosis of all patients and overcome the adverse prognostic impact of DMRGM.

### Efficacy of hypomethylating agents for patients with DMRGM

All but three patients with DMRGM were evaluated for the efficacy of induction therapy in our study (Additional file 1: Table S4). The CR/CRi after first induction of patients with DMRGM who received standard first-line chemotherapy, low-intensity chemotherapy, and other chemotherapy groups were 65.0%, 59.6%, and 31.3% separately. We performed a subgroup analysis in the low-intensity chemotherapy group based on whether HMA was combined. Intriguingly, we found that combining HMA with low-intensity chemotherapy increased the ORR of patients from 68.2% to 77.2%. Though it did not reach a statistically difference, a convincing trend was present which needed to be confirmed by future studies.

### External validation of the BeatAML database

The BeatAML database was used for external validation, consisting of 672 primary specimens from 562 patients with AML. Patients receiving WES at initial diagnosis were the main inclusion criteria. Duplications and incomplete records were excluded. Overall, 484 patients were finally enrolled after applying the inclusion and exclusion criteria. Next, we performed a 5:1 propensity score matching to confirm the consistency of enrolled patients according to the age, gender, and 2017 ELN risk stratification shown in Additional file 1: Table S5. Of the 168 patients, 47 (28.0%) had DMRGM, including 37 (22.2%) with a single mutation (DMRGM-1), 9 (5.4%) with a double mutation (DMRGM-2), and 1 (0.6%) with a triple mutation (DMRGM-3), which is similar to our study. The results of univariate analyses of DMRGM for OS are shown in Additional file 1: Figure S3. Patients with DMRGM experienced a poor OS (P < 0.001). In addition, a significant association between the number of DMRGM and OS was also found in BeatAML cohorts (P < 0.05), which supported our conclusion.

## Discussion

According to the results of this study, DMRGM was a frequent mutation set, occurring in 250 of 843 patients (29.7%). NPM1, FLT3-ITD, and FLT3-TKD were the three most frequently co-occurring mutated genes with DMRGM. There was an association between DMRGM and poor prognosis in both the present study and the BeatAML database. Furthermore, OS was negatively correlated with the number of DMRGM. The potential utility of a combination of HMAs merits consideration for patients deemed unsuitable for intensive induction therapy. Further investigation is warranted to validate this approach for clinical use. In addition, receiving HSCT significantly improved the prognosis of patients with DMRGM, resulting in better OS and RFS.

DNA methylation is a form of chromatin modification that plays a crucial role in regulating the expression of epigenetic genes and determining cell identity. It is a reversible process of attaching methyl residues to the five-carbon position of cytosine adjacent to guanine (CpGs), and the whole process is catalyzed by DNA methyltransferases (DNMTs), DNMT1, DNMT3A, and DNMT3B [25]. Several studies have shown that DNA methylation is intimately involved in normal biological functions such as stem cell self-renewal and immune cell differentiation [12, 26, 27]. Our previous study found that low-dose decitabine, a demethylating agent, increased platelet counts in transplant recipients with refractory prolonged isolated thrombocytopenia [28]. Emerging evidence suggests that dysregulated DNA methylation is a critical event in the initiation and progression of AML [11). However, some authors argue that CpG island hypermethylation is thought to be a consequence of rapid cell proliferation rather than a pathogenic event in AML development [29].

In AML, DNMT3A^R882H^ is a hotspot mutation causing aberrant DNA methylation [30]. It was shown that 44% of the original AML specimens in the Cancer Genome Atlas (TCGA) database contained nonsynonymous DNA methylation-associated mutations [7]. More importantly, IDH1/2 is a group of homologous enzymes that play an essential role in the tricarboxylic acid cycle and lipid metabolism. IDH1/2 mutations lead to abnormal accumulation of the “parametabolite” 2-HG and may inhibit TET2 function, dysregulate DNA methylation, and impair hematopoiesis [31]. Regarding the controversial results of TET mutations on prognosis, Liu WJ et al. performed a meta-analysis including 2552 patients who concluded that TET2 mutations appear to be a poor prognostic indicator in both cytogenetically normal (CN)-AML patients and in a subgroup of patients with favorable/moderate class I risk genotypes [32]. Rather than focusing on single or isolated DNA methylation-related mutations, we took these four DMRGMs as a whole and covered a large number of cases (n = 843), together with an external validation which made the results more convincing.

Furthermore, our results showed that OS was negatively correlated with the number of DMRGMs. Previous studies have reported that an increased number of driver mutations indicates a poorer prognosis for hematologic malignancies [33, 34]. An association between the number of oncogenic mutations and leukemia-free survival (LFS) was also found in MDS patients [35]. The transformation from MDS to AML may be driven by clonal evolution associated with acquisition of new driver mutations. These findings suggest that the accumulation of driver gene mutation interactions produces the leukemic phenotype. The accumulation of mutations is attributed to the genome-wide instability of AML. Therefore, attention should be paid not only to single-gene mutations but also to functionally similar subgroups of genes.

HMA, which mainly includes decitabine and azacitidine, has been widely used as standard treatment for patients with high-risk MDS or AML patients who are not suitable for intensive chemotherapy and are older [24, 36]. The current results show that low-intensity chemotherapy combined with HMA may help achieve a good treatment outcome for patients who are unsuitable for intensive induction therapy. Although it did not reach a statistically difference, a convincing trend was present. A multicenter, randomized phase III trial evaluated the efficacy of decitabine, and older patients receiving decitabine had better survival than low-dose cytarabine (median OS 7.7 vs. 5.0 months) [37]. Another clinical trial showed higher one-year survival with azacitidine than with the usual care regimen (46.5% vs. 34.2%) [38]. HMA seems to yield clinical benefits, and the specific molecular mechanisms of DNA demethylation and their therapeutic potential need to be further explored.

A pivotal point to consider in this study is the selection bias of single-center retrospective studies. Given the critical impact of MRD on the survival of AML patients and the large body of evidence on the association of DMRGM with pre-leukemic clones, it is a limitation that MRD was not included in the analysis of this study. Another limitation of this study was the small sample size involved in chemotherapy to explore the HMA on patients with DMRGM. Further multicenter and large sample whole genome sequencing or bisulfite sequencing studies are needed to validate our results and explore the potential molecular basis of DNA methylation.

## Conclusion

Our study provided an overview of AML patients with DMRGM and it was established as a risk factor for poor prognosis.

## Supplementary Information

**Additional file 1**. Supplementary figures and tables.

## Data Availability

The datasets used or analyzed during the current study are available from the corresponding author on reasonable request.

## Abbreviations

AML: Acute myeloid leukemia
NGS: Next-generation sequencing
MDS: Myelodysplastic syndromes
DNMT3A: DNA methyltransferase 3α
IDH1: Isocitrate dehydrogenase 1
IDH2: Isocitrate dehydrogenase 2
TET2: Tet methylcytidine dioxygenase 2
2017 ELN: 2017 European Leukemia Network
MRD: Minimal residual disease
DMRGM: DNA methylation regulatory gene mutations
HMA: Hypomethylating agents
HSCT: Hematopoietic stem cell transplantation
CR: Complete remission
CRi: CR with incomplete hematologic recovery
PR: Partial remission
ORR: Overall response rate
ATG: Anti-thymocyte globulin
OS: Overall survival
RFS: Relapse-free survival
TCGA: The Cancer Genome Atlas
CN: Cytogenetically normal
LFS: Leukemia-free survival
