# Supplementary material for: Clinical characteristics and prognostic significance of DNA methylation regulatory gene mutations in acute myeloid leukemia
Source: Clin Epigenetics. 2023 Mar 29;15:54. doi: 10.1186/s13148-023-01474-0 (PMC10061765; doi:10.1186/s13148-023-01474-0)
Supplement: Supplementary file 1 — Additional file 1. Supplementary figures and tables. [file 13148_2023_1474_MOESM1_ESM.docx]

**Additional file**

**Additional figure legends**

**Figure S1.** Overall survival (OS) and relapse-free survival (RFS) in AML with DMRGM-2 among different combinations. (a) OS of patients with DMRGM-2 among different combinations. (b) RFS of patients with DMRGM-2 among different combinations.

**Figure S2.** Overall survival (OS) and relapse-free survival (RFS) in AML patients based on different consolidation therapy. (a) OS in all patients based on different consolidation therapy. (b) RFS in all patients based on different consolidation therapy.

**Figure S3.** Overall survival (OS) in patients from BeatAML dataset**.** (a) OS in patients from BeatAML dataset based on DMRGM. (b) OS in patients from BeatAML dataset with different number of DMRGM.

**Table S1.** Pairwise comparison among multiple groups after Chi-square test

**Table S2.** Clinical characteristics of patients with DMRGM

**Table S3.** Relationship among each mutation with DMRGM

**Table S4.** Comparison of efficacy among different chemotherapy regimens

**Table S5.** Propensity score–matched cohort in BeatAML


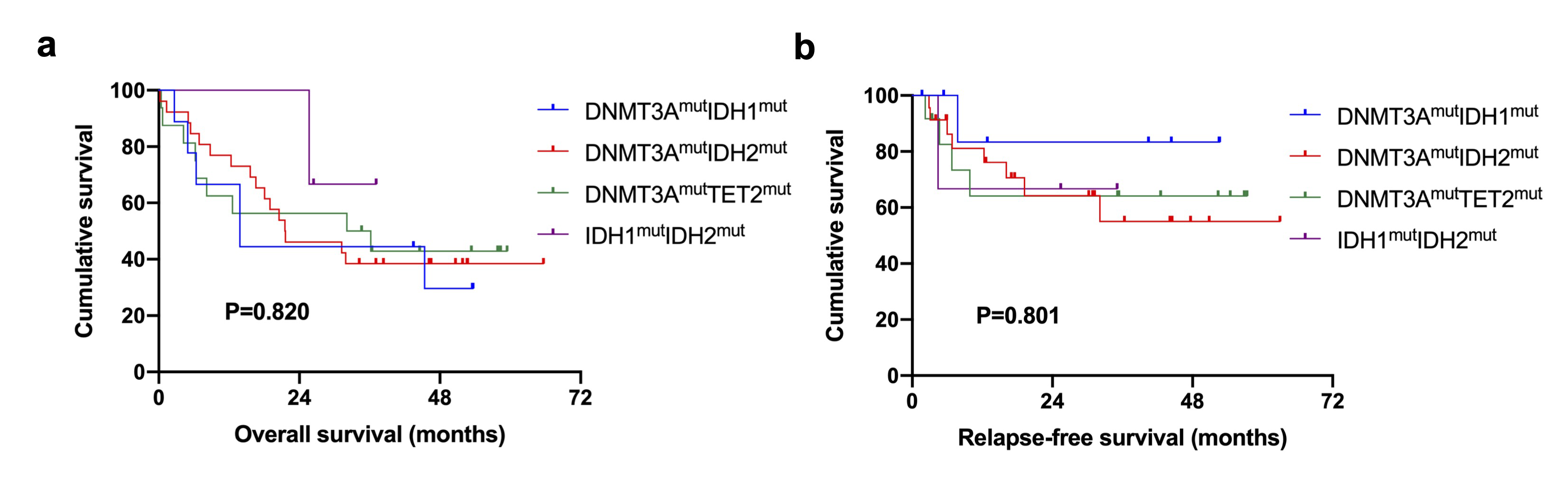


**Supplemental Figure 1.** (a) overall survival (OS) of patients with DMRGM-2 among different combinations. (b) relapse-free survival (RFS) of patients with DMRGM-2 among different combinations.

**
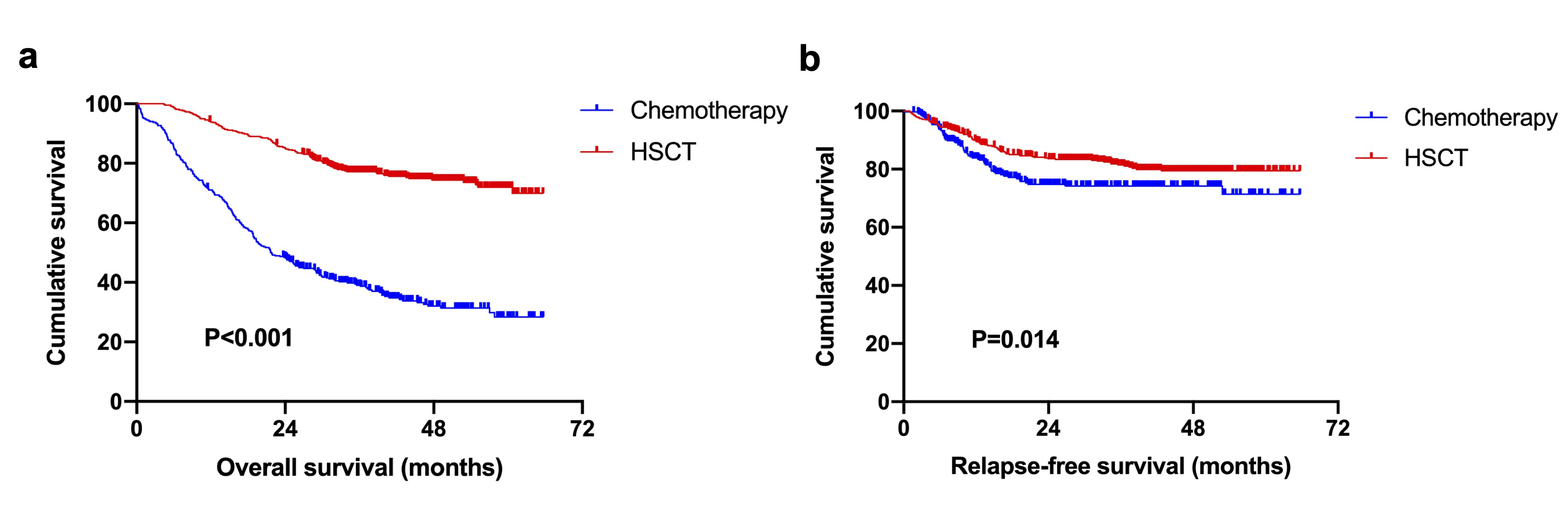
**

**Supplemental Figure 2.** (a) Overall survival (OS) in all patients based on different consolidation therapy. (b) Relapse-free survival (RFS) in all patients based on different consolidation therapy.


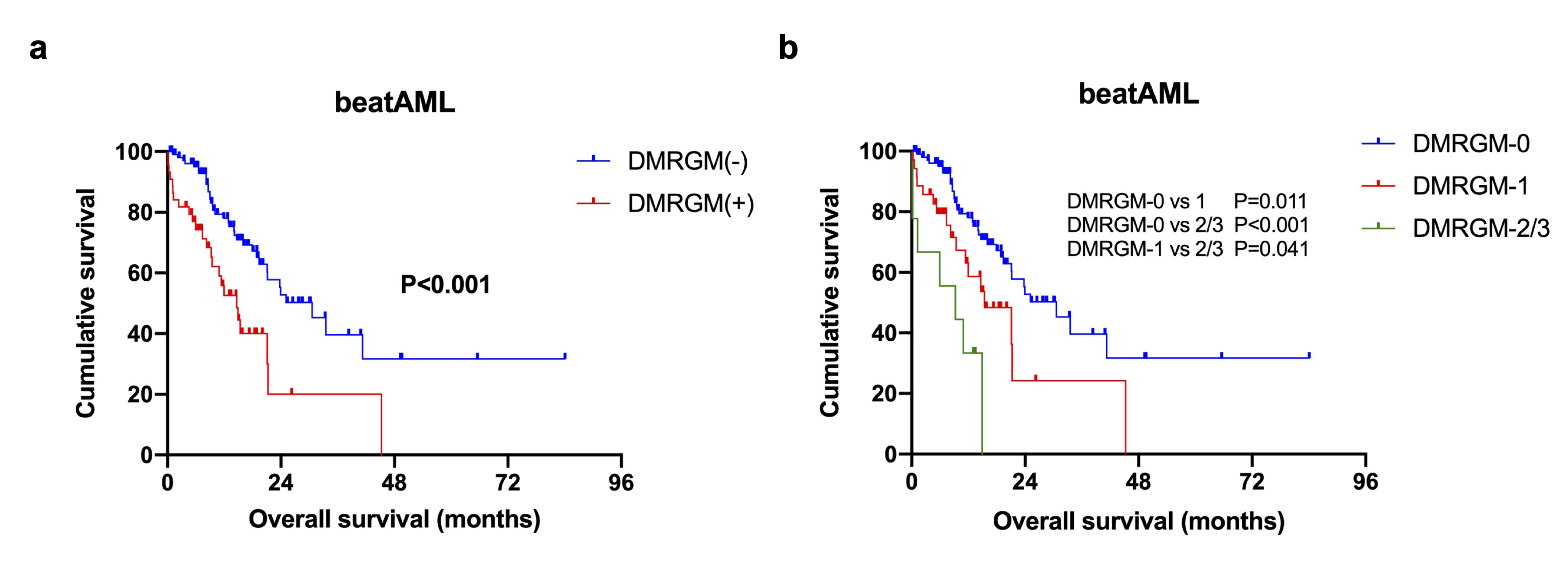


**Supplemental Figure 3.** (a) Overall survival (OS) in patients from BeatAML dataset based on DMRGM. (b) OS in patients from BeatAML dataset with different number of DMRGM.

**Supplemental Table 1.** Pairwise comparison among multiple groups after Chi-square test

| **Variables** | **DMRGM (+) n=250 (%)** | **DMRGM (-) n=593 (%)** |
| --- | --- | --- |
| **Karyotype** |  |  |
| Favorable | 8a (3.2) | 94a (15.9) |
| Intermediate | 208b, c (83.2) | 409b, c (69.0) |
| Adverse | 19a, c (7.6) | 72a, c (12.1) |
| Unknown | 15b (6.0) | 18b (3.0) |
| **2017 ELN** |  |  |
| Favorable | 99a, b (39.6) | 258a, b (43.5) |
| Intermediate | 87b (34.8) | 149b (25.1) |
| Adverse | 64a (25.6) | 186a (31.4) |
| **Induction therapy** |  |  |
| Standard therapy | 117a (46.8) | 360a (60.7) |
| Low intensity | 114b (45.6) | 194b (32.7) |
| Others | 19a, b (7.6) | 39a, b (6.6) |
| **Response to first induction therapy** |  |  |
| CR/CRi | 149a (60.3) | 417a (71.0) |
| PR | 33a, b (13.4) | 69a, b (11.8) |
| NR | 58b (23.5) | 89b (15.2) |
| Early death | 7a, b (2.8) | 12a, b (2.0) |

DMRGM: DNA methylation regulatory gene mutations; 2017 ELN: 2017 European Leukemia Network; CR: complete remission; CRi: complete remission with incomplete hematological recovery; PR: partial remission; NR: no response. ^a^Response to first induction therapy was unknown in 9 patients. Each subscript letter denotes a subset of work categories whose column proportions do not differ significantly from each other at the 0.05 level.

**Supplemental Table 2.** Clinical characteristics of patients with DMRGM

| **Variables** | **DNMT3A** | |  | **IDH1** | |  | **IDH2** | |  | **TET2** | |
| --- | --- | --- | --- | --- | --- | --- | --- | --- | --- | --- | --- |
|  | **n=109 (%)** | **P value** |  | **n=41(%)** | **P value** |  | **n=78 (%)** | **P value** |  | **n=84 (%)** | **P value** |
| **Gender** |  | 0.128 |  |  | 0.609 |  |  | 0.487 |  |  | 0.956 |
| Male | 50(45.9) |  |  | 20(48.8) |  |  | 44(56.4) |  |  | 44(52.4) |  |
| Female | 59(54.1) |  |  | 21(51.2) |  |  | 34(43.6) |  |  | 40(47.6) |  |
| **Age, years** |  | ***<0.001*** |  |  | ***0.002*** |  |  | ***<0.001*** |  |  | 0.205 |
| Median (Range) | 52(19-78) |  |  | 50(24-71) |  |  | 50(17-75) |  |  | 47(10-71) |  |
| **WBC count, *10^9^/L** | | ***<0.001*** |  |  | ***0.002*** |  |  | 0.931 |  |  | ***0.007*** |
| Median (Range) | 46.3(0.6-383.8) |  |  | 6.9(0.6-275.0) |  |  | 11.9(0.5-331.2) |  |  | 23.3(0.8-383.8) |  |
| **Hemoglobin, g/L** | | 0.355 |  |  | 0.079 |  |  | 0.054 |  |  | 0.973 |
| Median (Range) | 86(38-136) |  |  | 94(54-140) |  |  | 76(35-139) |  |  | 87(34-136) |  |
| **Platelet count, *10^9^/L** | | ***<0.001*** |  |  | ***<0.001*** |  |  | ***0.010*** |  |  | 0.441 |
| Median (Range) | 63(11-713) |  |  | 77(4-713) |  |  | 52(5-508) |  |  | 45(5-303) |  |
| **Karyotype** |  | ***<0.001*** |  |  | ***0.034*** |  |  | ***<0.001*** |  |  | 0.371 |
| Favorable | 0(0.0) |  |  | 0(0.0) |  |  | 2(2.6) |  |  | 6(7.1) |  |
| Intermediate | 96(88.1) |  |  | 37(90.2) |  |  | 63(80.8) |  |  | 67(79.8) |  |
| Adverse | 7(6.4) |  |  | 4(9.8) |  |  | 4(5.1) |  |  | 7(8.3) |  |
| Unknown | 6(5.5) |  |  | 0(0.0) |  |  | 9(11.5) |  |  | 4(4.8) |  |
| **2017 ELN** |  | ***0.004*** |  |  | 0.452 |  |  | 0.052 |  |  | 0.751 |
| Favorable | 38(34.9) |  |  | 15(36.6) |  |  | 27(34.6) |  |  | 38(45.2) |  |
| Intermediate | 45(41.3) |  |  | 15(36.6) |  |  | 31(39.7) |  |  | 24(28.6) |  |
| Adverse | 26(23.9) |  |  | 11(26.8) |  |  | 20(25.6) |  |  | 22(26.2) |  |
| **Induction therapy** | | ***0.005*** |  |  | 0.134 |  |  | ***0.011*** |  |  | 0.416 |
| Standard therapy | 48(44.0) |  |  | 18(43.9) |  |  | 32(41.0) |  |  | 42(50.0) |  |
| Low intensity | 55(50.5) |  |  | 21(51.2) |  |  | 40(51.3) |  |  | 35(41.7) |  |
| Others | 6(5.5) |  |  | 2(4.9) |  |  | 6(7.7) |  |  | 7(8.3) |  |
| **Response to first induction therapy^a^** | | ***0.007*** |  |  | 0.824 |  |  | ***0.007*** |  |  | 0.419 |
| CR/CRi | 61(56.0) |  |  | 30(73.2) |  |  | 40(52.6) |  |  | 50(60.2) |  |
| PR | 13(11.9) |  |  | 5(12.2) |  |  | 10(13.2) |  |  | 13(15.7) |  |
| NR | 31(28.4) |  |  | 5(12.2) |  |  | 24(31.6) |  |  | 17(20.5) |  |
| Early death | 4(3.7) |  |  | 1(2.4) |  |  | 2(2.6) |  |  | 3(3.6) |  |
| **Transplant** |  | ***0.033*** |  |  | 0.734 |  |  | 0.160 |  |  | 0.775 |
| Yes | 51(46.8) |  |  | 22(53.7) |  |  | 38(48.7) |  |  | 46(54.8) |  |
| No | 58(53.2) |  |  | 19(46.3) |  |  | 40(51.3) |  |  | 38(45.2) |  |

DMRGM: DNA methylation regulatory gene mutations; WBC: white blood cell; 2017 ELN: 2017 European Leukemia Network; CR: complete remission; CRi: complete remission with incomplete hematological recovery; PR: partial remission; NR: no response. ^a^Response to first induction therapy was unknown in 2 patients with IDH2 mutation and 1 patient with TET2 mutation. A P value of less than 0.05 is indicated in italics and bold.

**Supplemental Table 3.** Relationship among each mutation with DMRGM

| **Gene mutation** | **DNMT3A** | |  | **IDH1** | |  | **IDH2** | |  | **TET2** | |  | **DMRGM** | |
| --- | --- | --- | --- | --- | --- | --- | --- | --- | --- | --- | --- | --- | --- | --- |
|  | **n=109 (%)** | **P value** |  | **n=41**  **(%)** | **P value** |  | **n=78 (%)** | **P value** |  | **n=84 (%)** | **P value** |  | **n=250 (%)** | **P value** |
| **FLT3-ITD** | 37(33.9) | ***<0.001*** |  | 7(17.1) | 0.855 |  | 12(15.4) | 0.506 |  | 21(25.0) | 0.086 |  | 64(25.6) | ***<0.001*** |
| **NPM1** | 58(53.2) | ***<0.001*** |  | 18(43.9) | ***<0.001*** |  | 28(35.9) | ***<0.001*** |  | 31(36.9) | ***<0.001*** |  | 108(43.2) | ***<0.001*** |
| **DNMT3A** | —— | —— |  | 11(26.8) | ***0.007*** |  | 28(35.9) | ***<0.001*** |  | 18(21.4) | ***0.014*** |  | —— | —— |
| **CEBPAbm** | 7(6.4) | ***0.032*** |  | 0(0.0) | ***0.012*** |  | 3(3.8) | ***0.013*** |  | 10(11.9) | 0.793 |  | 19(7.6) | ***0.003*** |
| **NRAS** | 12(11.0) | 0.862 |  | 4(9.8) | 0.719 |  | 8(10.3) | 0.716 |  | 6(7.1) | 0.187 |  | 21(8.4) | 0.066 |
| **TET2** | 18(16.5) | ***0.014*** |  | 4(9.8) | 0.964 |  | 2(2.6) | ***0.022*** |  | —— | —— |  | —— | —— |
| **KIT** | 2(1.8) | ***0.003*** |  | 0(0.0) | ***0.030*** |  | 5(6.4) | 0.285 |  | 5(6.0) | 0.207 |  | 11(4.4) | ***0.001*** |
| **WT1** | 7(6.4) | 0.242 |  | 1(2.4) | 0.114 |  | 5(6.4) | 0.330 |  | 7(8.3) | 0.703 |  | 19(7.6) | 0.224 |
| **IDH2** | 28(25.7) | ***<0.001*** |  | 2(4.9) | 0.322 |  | —— | —— |  | 2(2.4) | ***0.022*** |  | —— | —— |
| **FLT3-TKD** | 18(16.5) | ***0.001*** |  | 8(19.5) | ***0.009*** |  | 9(11.5) | 0.298 |  | 7(8.3) | 0.975 |  | 33(13.2) | ***0.001*** |
| **RUNX1** | 13(11.90 | 0.050 |  | 4(9.8) | 0.546 |  | 14(17.9) | ***<0.001*** |  | 3(3.6) | 0.162 |  | 26(10.4) | ***0.028*** |
| **GATA2** | 3(2.8) | 0.080 |  | 1(2.4) | 0.268 |  | 1(1.3) | ***0.046*** |  | 10(11.9) | ***0.041*** |  | 14(5.6) | 0.430 |
| **KRAS** | 5(4.6) | 0.462 |  | 4(9.8) | 0.328 |  | 2(2.6) | 0.165 |  | 2(2.4) | 0.128 |  | 10(4.0) | 0.089 |
| **ASXL1** | 3(2.8) | 0.112 |  | 3(7.3) | 0.754 |  | 6(7.7) | 0.557 |  | 8(9.5) | 0.178 |  | 17(6.8) | 0.621 |
| **PTPN11** | 10(9.2) | 0.056 |  | 5(12.2) | ***0.045*** |  | 3(3.8) | 0.538 |  | 0(0.0) | ***0.022*** |  | 12(4.8) | 0.652 |
| **JAK** | 2(1.8) | 0.089 |  | 2(4.9) | 0.920 |  | 7(9.0) | 0.118 |  | 2(2.4) | 0.218 |  | 10(4.0) | 0.301 |
| **IDH1** | 11(10.1) | ***0.007*** |  | —— | —— |  | 2(2.6) | 0.322 |  | 4(4.8) | 0.964 |  | —— | —— |

DMRGM: DNA methylation regulatory gene mutations. A P value of less than 0.05 is indicated in italics and bold.

**Supplemental Table 4.** Comparison of efficacy among different chemotherapy regimens

| **Response to first induction therapy** | **AML with DMRGM**  **(n=247)** | | | |  | | **AML with DMRGM receiving low-intensity chemotherapy (n=114)** | | | |
| --- | --- | --- | --- | --- | --- | --- | --- | --- | --- | --- |
|  |  |  |  |  |  | |  |  |  |  |
|  | **Standard therapy** | **Low intensity** | **Others** | **P value** | |  | | **without HMA** | **with HMA** | **P value** |
| CR/CRi | 76(65.0) | 68(59.6) | 5(31.3) | ***<0.001*** | |  | | 13(59.1) | 55(59.8) | 0.164 |
| PR | 12(10.3) | 18(15.8) | 3(18.8) |  | |  | | 2(9.1) | 16(17.4) |  |
| NR | 27(23.1) | 27(23.7) | 4(25.0) |  | |  | | 6(27.3) | 21(22.8) |  |
| Early death | 2(1.7) | 1(0.9) | 4(25.0) |  | |  | | 1(4.5) | 0(0.0) |  |

Response to first induction therapy was unknown in 3 patients with DMRGM. CR: complete remission; CRi: complete remission with incomplete hematological recovery; PR: partial remission; NR: no response. DMRGM: DNA methylation regulatory gene mutations. A P value of less than 0.05 is indicated in italics and bold.

**Supplemental Table 5.** Propensity score–matched cohort in BeatAML

| **Variables** | **Before matching** | |  |  | **After matching (5:1)** | |  |
| --- | --- | --- | --- | --- | --- | --- | --- |
|  | **This study n=843 (%)** | **BeatAML n=484 (%)** | **P value** |  | **This study n=843 (%)** | **BeatAML n=168(%)** | **P value** |
| **Gender** |  |  | 0.380 |  |  |  | 0.790 |
| Male | 444(52.7) | 267(55.2) |  |  | 444(52.7) | 86(51.2) |  |
| Female | 399(47.3) | 217(44.8) |  |  | 399(47.3) | 82(48.8) |  |
| **Age, years** |  |  | ***<0.001*** | |  |  | 0.304 |
| Median (Range) | 43(9-78) | 62(1-87) |  |  | 43(9-78) | 45(8-81) |  |
| **2017 ELN** |  |  | ***<0.001*** | |  |  | 0.231 |
| Favorable | 357(42.3) | 132(27.3) |  |  | 357(42.3) | 60(35.7) |  |
| Intermediate | 236(28.0) | 162(33.5) |  |  | 236(28.0) | 49(29.2) |  |
| Adverse | 250(29.7) | 190(39.3) |  |  | 250(29.7) | 59(35.1) |  |

2017 ELN: 2017 European Leukemia Network
